# Reciprocity in the Developmental Regulation of Aquaporins 1, 3 and 5 during Pregnancy and Lactation in the Rat

**DOI:** 10.1371/journal.pone.0106809

**Published:** 2014-09-03

**Authors:** Sasan Nazemi, Mette Rahbek, Ladan Parhamifar, Seyed Moein Moghimi, Hamid Babamoradi, Foojan Mehrdana, Dan Arne Klærke, Christopher H. Knight

**Affiliations:** 1 Department of Veterinary Clinical and Animal Sciences (IKVH) Faculty of Health and Medical Sciences, University of Copenhagen, Copenhagen, Denmark; 2 Centre for Pharmaceutical Nanotechnology and Nanotoxicology, Department of Pharmacy, Faculty of Health and Medical Sciences, University of Copenhagen, Copenhagen, Denmark; 3 Department of Food Sciences, Spectroscopy and Chemometrics section, Faculty of Science, University of Copenhagen, Copenhagen, Denmark; 4 Department of Veterinary Disease Biology (IVS), Parasitology and Aquatic Diseases, Faculty of Health and Medical Sciences, University of Copenhagen, Copenhagen, Denmark; University of Bari Aldo Moro, Italy

## Abstract

Milk secretion involves significant flux of water, driven largely by synthesis of lactose within the Golgi apparatus. It has not been determined whether this flux is simply a passive consequence of the osmotic potential between cytosol and Golgi, or whether it involves regulated flow. Aquaporins (AQPs) are membrane water channels that regulate water flux. AQP1, AQP3 and AQP5 have previously been detected in mammary tissue, but evidence of developmental regulation (altered expression according to the developmental and physiological state of the mammary gland) is lacking and their cellular/subcellular location is not well understood. In this paper we present evidence of developmental regulation of all three of these AQPs. Further, there was evidence of reciprocity since expression of the rather abundant AQP3 and less abundant AQP1 increased significantly from pregnancy into lactation, whereas expression of the least abundant AQP5 decreased. It would be tempting to suggest that AQP3 and AQP1 are involved in the secretion of water into milk. Paradoxically, however, it was AQP5 that demonstrated most evidence of expression located at the apical (secretory) membrane. The possibility is discussed that AQP5 is synthesized during pregnancy as a stable protein that functions to regulate water secretion during lactation. AQP3 was identified primarily at the basal and lateral membranes of the secretory cells, suggesting a possible involvement in regulated uptake of water and glycerol. AQP1 was identified primarily at the capillary and secretory cell cytoplasmic level and may again be more concerned with uptake and hence milk synthesis, rather than secretion. The fact that expression was developmentally regulated supports, but does not prove, a regulatory involvement of AQPs in water flux through the milk secretory cell.

## Introduction

It has been proposed that mammary glands evolved as a specialised component of the immune system [1], [2], acquiring the ability to synthesize specific milk components and secrete them together with copious amounts of water. One of the specific milk components is lactose, a complex carbohydrate unique to the mammary gland and synthesized, again uniquely, within the Golgi apparatus. Its precursors (glucose and galactose) can diffuse readily across the Golgi membrane but lactose cannot, hence, it builds up inside the vacuole and provides an osmotic drive for water to enter the Golgi [3]. In common with the other specific milk constituents (casein, milk fat) the regulation of lactose synthesis and secretion has been extensively researched. On the other hand, the precise way in which water moves into the Golgi and hence into milk has received very little attention. Since the concentration of lactose in milk is generally rather constant within any given species, it has commonly been assumed that the water flux is passive. However, there is a major exception to this rule. The composition of marsupial milk changes quite dramatically during the course of lactation. Early lactation (Phases 1, 2a and 2b in the terminology of Nicholas, 1988 [4]) is characterised by relatively sparse secretion of milk that is high in lactose, whereas later lactation (Phase 3) comprises copious secretion of low-lactose milk. It seems inevitable that, under these circumstances, something other than lactose secretion must be regulating water flux. Aquaporins (AQP) are regulated, channel-forming membrane proteins that transport water (and, in some cases, other small solutes; see Carbrey and Agre, 2009 for review [5]). So far, 13 AQP have been found [6], [7], characterization of 6 of these (AQP 0–5) is well advanced due to their higher tissue expression and better availability of antibodies [8] and AQP1, AQP3 and AQP5 have been shown to have significant levels of mRNA expression in a number of different tissues [9] AQP are divided into groups based on their permeabilities. The aquaglyceroporins (AQP3, AQP7, AQP9 and AQP10) transfer glycerol in addition to water and urea, whilst APQ6 and AQP8 (orthodox aquaporins) largely transport anions and permeate ammonia (and water), respectively. AQP11 and AQP12 are known as super-aquaporins on account of their relatively low sequence homology with other AQP [10]. Recently an important role in cell organelle function was linked to these AQPs, which are suggested to transport water within the cell [11]. The remaining AQP permeate water. The first evidence of AQP expression in mammary tissue was made in mammary carcinomas [12] but more recently expression of AQP1, AQP3 and AQP5 has been demonstrated in normal mammary tissue of rodents and cattle [13], [14]. However, little is known about the developmental regulation and functionality of mammary AQP during pregnancy and lactation. Lactogenesis stage II, the initiation of copious milk secretion at or around parturition [15] marks a rapid and precipitous transition (within 24 h) from minimal water flux through the mammary gland to very significant flux (many litres in dairy species), hence, once could anticipate a major increase in mammary AQP expression at this time. Furthermore, the ability of certain AQP to transport glycerol suggests a second potential role, since the mammary gland synthesizes large amounts of triglyceride and so has a significant requirement for glycerol. The present study, therefore, investigates expression level and localization of AQP1, AQP3 and AQP5 in rat mammary tissue during pregnancy and two stages of lactation (early lactation and maximal secretion, or peak lactation) using qRT-PCR, Western blotting and immunohistochemistry.

## Materials and Methods

### Animals

The experiment was performed according to the guidelines of the European Convention for the Protection of Vertebrate Animals and Animal Experimentation Act under Danish national legislation. All the experimental protocols were approved by The Council for Animal Experimentation of the Danish Ministry of Food, Agriculture and Fisheries at Danish Veterinary and Food Administration (permit number: 2012-15-2934-00587) and conducted at the Danish Technical University (Denmark). All efforts were made to minimize suffering. Nine pregnant female (between 4–6 days into pregnancy) Sprague-Dawley rats of 270–310 g were obtained from Taconic (Denmark). Rats were caged in single cages and had free access to breeding diet and water. They were randomly assigned into group of three, and then euthanized by sodium pentobarbital intraperitoneal (Mebumal SAD 50 mg/ml, Denmark) injection of 200 mg/kg at different stages: a) pregnant 14±2 days within pregnancy, b) early lactation 4±1 days within lactation and c) peak lactation 14±1 days within lactations. Numbers of pups were adjusted to 10 for each lactating mother in order to minimize the effects of number of pups on lactation.

### Sampling

Mammary glands from the abdominal region were rapidly removed after euthanasia, divided into pieces for flash freezing in liquid nitrogen and stored at −80°C. Some parts of the tissues were transferred into 4% PFA (v/v) (paraformaldehyde) for 24 hours and then placed in 70% ethanol for 3 days before paraffin embedding.

### RNA preparation

Frozen mammary glands were cut into smaller pieces of 35–45 mg on dry ice, homogenized with 650 µl Trizol (Life Technologies Europe BV, 2850 Naerum, Denmark) by frozen stainless steel beads (5 mm Qiagen Cat.no 69989), in TissueLyser II from Qiagen (Cat.no 85300). Subsequently samples were centrifuged for 10 minutes at 4°C and the supernatant was transferred into MaXtract High Density 1.5 ml tubes (Qiagen Cat.no 129046) by using SV Total RNA isolation system kit from Promega (Naka, Sweden) according to the manufacturer’s instructions. RNA yield was then checked by NanoDrop ND-1000 Spectrophotometer and quality of the RNA was determined using Aligent 2100 Bioanalyzer System (Aligent Technologies).

### cDNA synthesis, primer design and qRT-PCR

Briefly, isolated RNA was transcribed to cDNA using 5x MMLV reverse transcriptase, dNTP, random hexamer primer and RNase inhibitor and MMLV enzyme by adding H_2_O up to 25 µl cocktail in total. cDNA was transcribed by G-STORM GS1. The program was: 25°C for 10 minutes, 42°C for 60 minutes and 95°C for 5 minutes (according to guideline). cDNA was stored at −20°C for short period and −80°C for longer times and also diluted into a concentration of 4 ng/µL before quantification. Primers were designed and blasted by NCBI nucleotide database to ensure specificity and analyzed by NetPrimer (online based software). Normfinder (free download software) was also used for identifying the optimal genes among these sets of candidates and ensuring their specificity. Three reference genes were selected and examined for gene expression analysis, ACTB (Beta-actin), TBP (TATA-binding protein) and GAPDH (glyceraldehyde 3-phosphate dehydrogenase). However, on the basis of the Normfinder analysis GAPDH was selected as reference gene for final normalization of the data.

Standard curves were obtained and acceptable efficiency range was set to 1.8–2 with satisfying melting curves. Samples were run in triplicate with inter-plate calibrators using LightCycler 480 machine (SYBr green 1 master mix) from Roche Applied and Diagnostics (Hvidovre, Denmark) 10 µM for each primer in a 10 µl reaction mixture containing 2 µl of total cDNA. The procedure consisted of an initial denaturation of the template at 94°C for 5 min, followed by 25 cycles of amplification (94°C for 1 min, 55°C for 1 min, 72°C for 1 min), and a final extension step at 72°C for 5 min. To ensure amplification of the correct sequence, PCR products were subjected to DNA-sequencing using a commercial service (TAG Copenhagen A/S). Primer sequences are listed in Table 1.

**Table 1:** Primer sequences used for real-time PCR reactions.

Gene	Sequence	Amplicon (bp)	NCBI ref sequence
AQP1		209	NM_012778.1
Forward	5′GACATGCAACAGACCGA 3′		
Reverse	5′AGAAGCCACAGCATCAGGTC 3′		
AQP3		337	NM_031703.1
Forward	5′TGGACCTCGCCTTTTCACTG 3′		
Reverse	5′GGAGCGTTTTTAGCCCGAGA 3′		
AQP5		253	NM_012779.1
Forward	5′ CTGGCGGCCATCCTCTATTT 3′		
Reverse	5′CCCCAGCTGAGAGGATGTTG 3′		
Gapdh		225	NM_017008.4
Forward	5′TGACAACTTTGGCATCGTGG 3′		
Reverse	5′ACATTGGGGGTAGGAACACG 3′		

### Western blotting

Rat mammary tissue (0.3 g) was homogenized in 300 µl lysis-buffer containing 20 mM HEPES, 2 mM MgCl_2_, 1 mM EDTA and 1 mM EDTA. Protein concentration was measured by Qubit (Fluorometric Quatification, Life Technologies). Equal amounts of protein, calculated based on protein concentration obtained from Qubit, was loaded in each well of a 4–12% BOLT Bis-Tris Plus Gels (Life Technologies) and subjected to electrophoresis in NuPAGE MES SDS running buffer (20X) (Life Technologies). Separated proteins were electrophoretically transferred onto polyvinylidene difluoride (PVDF) membrane by iBlot Dry Blotting System (Life Technologies) at 20 V for 7 min. Membranes were blocked in TBS buffer (137 mM NaCl and 20 mM Tris) with 0,1% Tween-20 and 5% skimmed milk. Anti-Aquaporin 1 (ab9566), 3(ab125219) and 5(ab78486) from Abcam company (UK) plus GAPDH (D16H11) Rabbit mAb (HRP conjugate) from Cell signaling (loading control); antibodies were purchased and recommended concentrations were used. Primary antibodies against indicated proteins were diluted 1∶1000 in TBS+0,1% Tween 20 and 3% BSA and incubated overnight at 4°C followed by three washes, each one for 5 min, in the TBS-Tween buffer. After washing three times for 5 minutes each time, the membranes were incubated for 1 hour at room temperature with 1∶5000 dilutions of HRP-conjugated secondary antibodies in 5% non-fat dry milk/TBST. The membranes were then washed as described above and incubated with ECL Western blot detection reagents (Life Technologies) and exposed for two min to Amersham Hyperfilm-ECL (VWR) to visualize immunoreactive proteins.

### Immunohistochemistry

Paraffin sections were obtained at 4 µm thickness using a microtome (Jung) and deparaffinized by heating in an oven for 25±5 minutes at 56°C followed by antigen retrieval (for AQP1 and AQP3 and according to specific requirements for each antibody) with microwave oven (sodium citrate 0.01 M, pH 6, at 90°C), blocking endogenous peroxidase in 3% H_2_O_2_ (Sigma Aldrich, Denmark) in methanol, then washing twice with PBS buffer. Non-specific binding of antibodies was blocked with 10% goat serum (Sigma Aldrich, Denmark) followed by overnight incubation with primary antibodies Anti-Aquaporin 1 [1∶100] (ab9566), Anti-Aquaporin 3 [1∶4500] (ab125219) and Anti-Aquaporin 5 [1∶1000] (ab78486).

Secondary antibodies were: polyclonal goat anti-rabbit (Dako, E0432) for AQP3 and AQP5 [1∶500] and polyclonal goat anti-mouse (Dako E0433) [1∶500]. After wash the sections were incubated with Streptavidin/HRP (DAKOP0397, 1∶500) in PBS for 30 minutes. Color reaction was developed with 3.3′-Diaminobenzidine (DAB) tablets/SigmaFAST (Sigma Alrdich, Denmark) and sections were counterstained with haematoxylin and eosin (H&E) before mounting in Glycergel (S3025, Dako). All the positive sections were accompanied in parallel with negative controls by excluding primary antibodies.

Iso-type controls were made using rabbit IgG (Dako, x0903) for AQP3 and AQP5 [1∶2860 and 1∶20000 respectively] and mouse IgG2b (Dako, x0944) for AQP1 [1∶10].

Statistical analysis for gene expression was performed based on [16] and using MATLAB 2012b (MathWorks) with one-way analysis of variance *(anova1)* and Tukey HSD test as a built-in function, the level of significance was set at P<0.05. For Western-blotting relative density of the protein was calculated by ImageJ software in comparison to GAPDH (as loading control) and graphs were made using SigmaPlot 12.0.

## Results

### mRNA expression of AQPs

**Figure 1:**
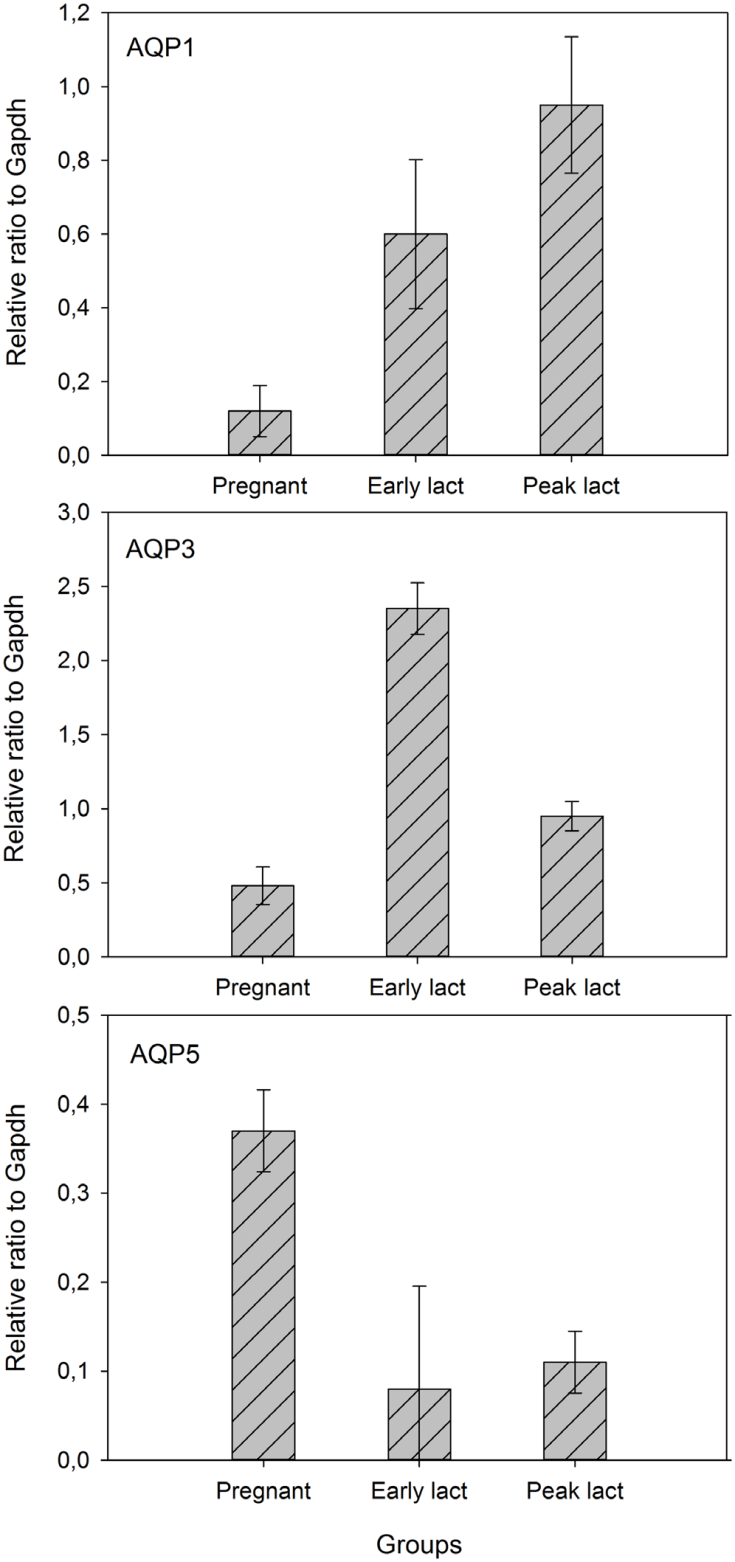
The relative expression of three different aquaporins. Gradual increase for AQP1 (a), high expression of AQP3 during early lactation (b) and gradual decrease for AQP5(c) during pregnancy, early lactation and peak lactation. Graphs presented as mean±SE.

Expression levels are shown in Fig. 1. AQP3 was most expressed, AQP1 was intermediate and AQP5 was least expressed. Significant developmental regulation was observed for all three. Expression of AQP1 and AQP3 increased from pregnancy to early lactation and then remained elevated (AQP1) or decreased (AQP3). However, expression of AQP5 was highest in pregnancy and then decreased and remained low. With the exception of the initial increase in AQP1, all changes were significant (Table 2).

**Table 2:** Expression of AQP1, AQP3 and AQP5 reported relative to expression of GAPDH.

	P	E	L
AQP1	0.12±0.12^a^	0.60±0.35^ab^	0.95±0.32^b^
AQP3	0.48±0.22^a^	2.35±0.30^b^	0.95±0.17^c^
AQP5	0.37±0.08^a^	0.08±0.20^bc^	0.11±0.06^c^

Results are mean ± SD and P-values from one-way ANOVA. Different letters within a row indicate significant difference between stages, where P = pregnant, E = early lactation, L = peak lactation.

### Western blot

**Figure 2:**
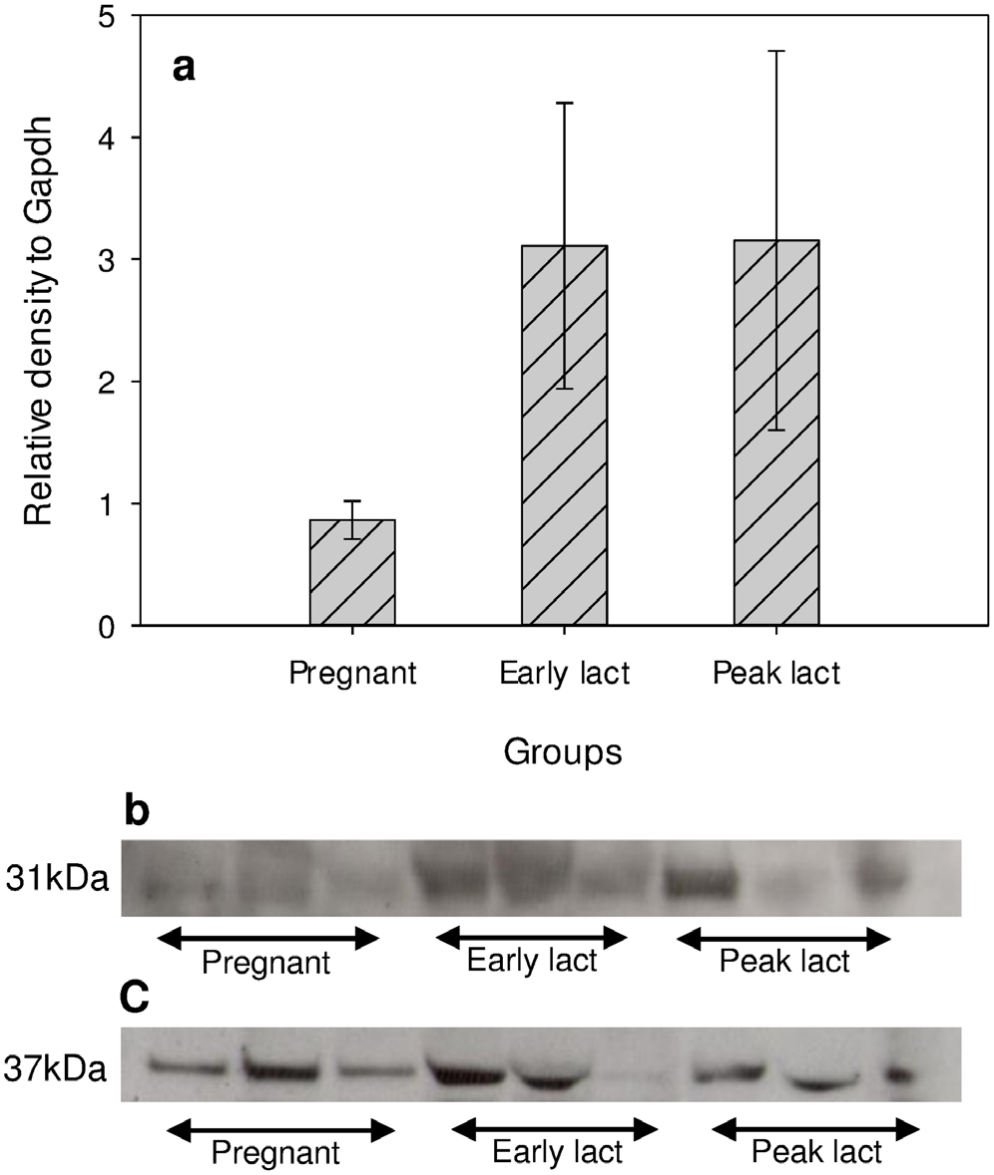
Protein expression of AQP3 (molecular weight of 31kDa: panel b) in pregnant, early lactation and peak lactation groups. Panel a) reports expression relative to GAPDH (molecular weight of 37 kDa: panel c) as mean ± SE. (0.86±0.15, 3.11±1.17 and 3.15±1.55 for pregnant, early lactation and peak lactation, respectively).

AQP3 was successfully visualized on X-ray films (Figure 2). Protein expression increased between pregnancy and early lactation but, in contrast to RNA expression, then remained elevated rather than decreasing. Despite a considerable amount of effort, AQP1 and AQP5 proteins were not detected in Western blots.

### Immunohistochemistry

**Figure 3:**
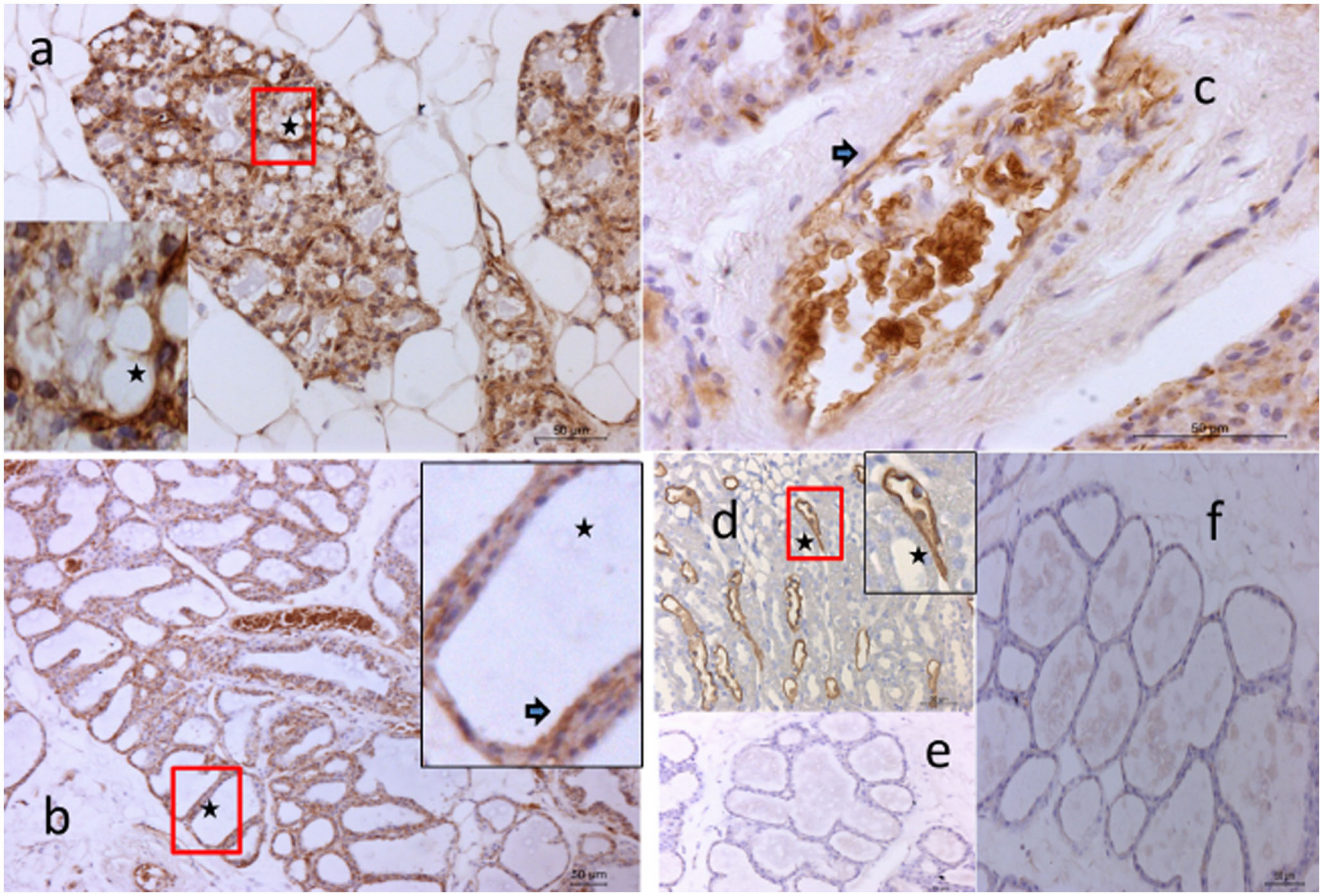
Immunostaining of rat mammary gland for AQP1. Panel a) shows pregnant tissue (10X) with (inset, 40X) developing alveoli showing AQP1 expression. Panel b) shows early lactation tissue (low magnification of secretory epithelium 10X and indicated by the arrow 40X). Panel c) Immuno staining of a large blood vessel (indicated by the arrow 40X) at peak lactation. Panel d) Positive control, collecting tubules of rat kidney (20X). Panel e) Negative control during early lactation (20X). Panel f) Isotype control, IgG2b during early lactation (20X).

**Figure 4:**
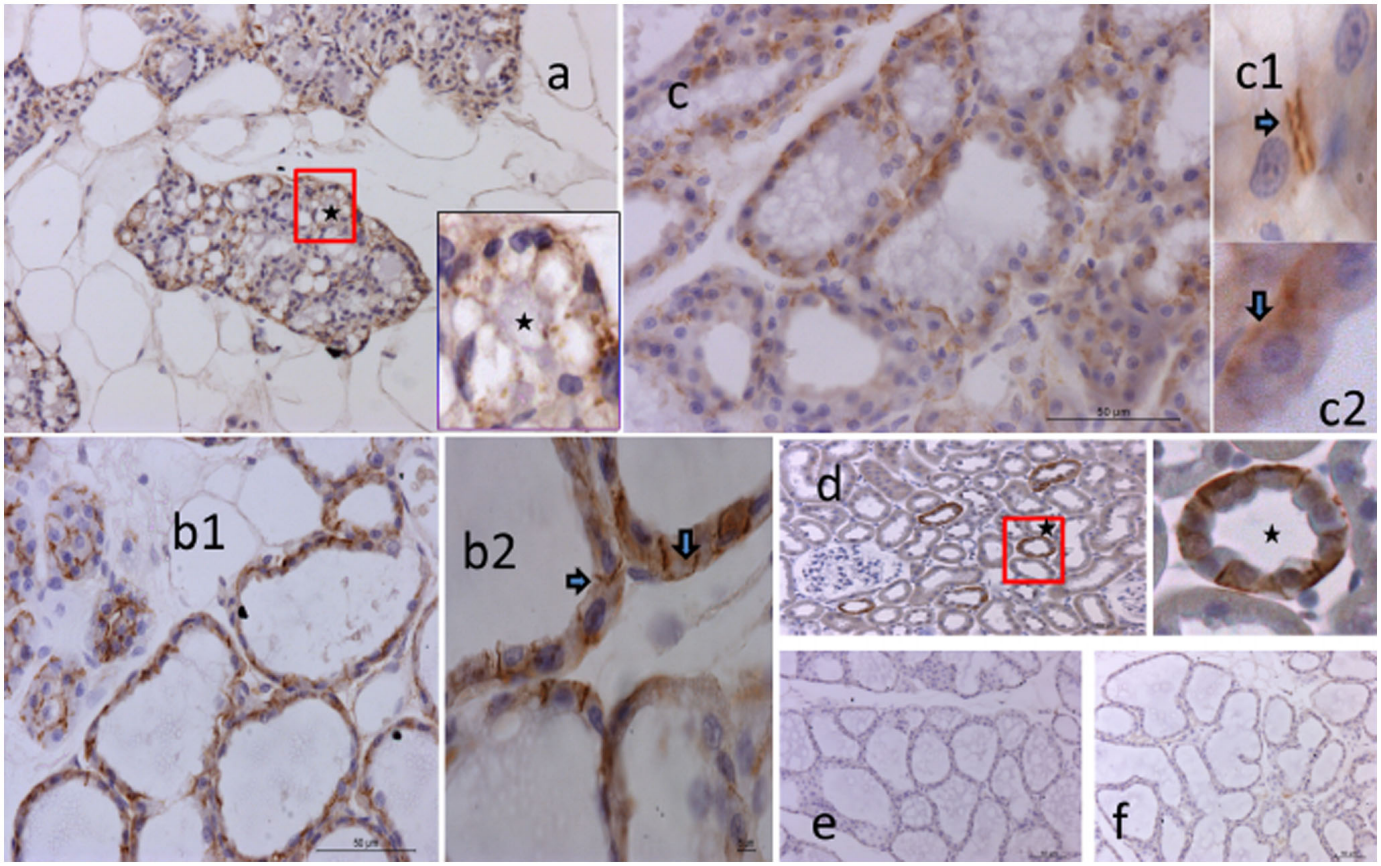
Immunostaining of rat mammary gland for AQP3 in three groups. Panel a) pregnant (developing alveoli 10X) Showing AQP3 expression. Panel b1) Immuno staining at early lactation 40X. Panel b2) showing signals in both at basal and lateral membrane with higher magnification (indicated by the arrows 100X). Panel c) Peak lactation 40X and Panel c1 and c2) immune staining with higher magnification (indicated by the arrows 100X) secretory epithelium both at basal and lateral membrane. Panel d) Positive control, proximal convoluted tubules of rat kidney (40X). Panel e) Negative control during early lactation (20X). Panel f) Isotype control, IgG during early lactation (20X).

**Figure 5:**
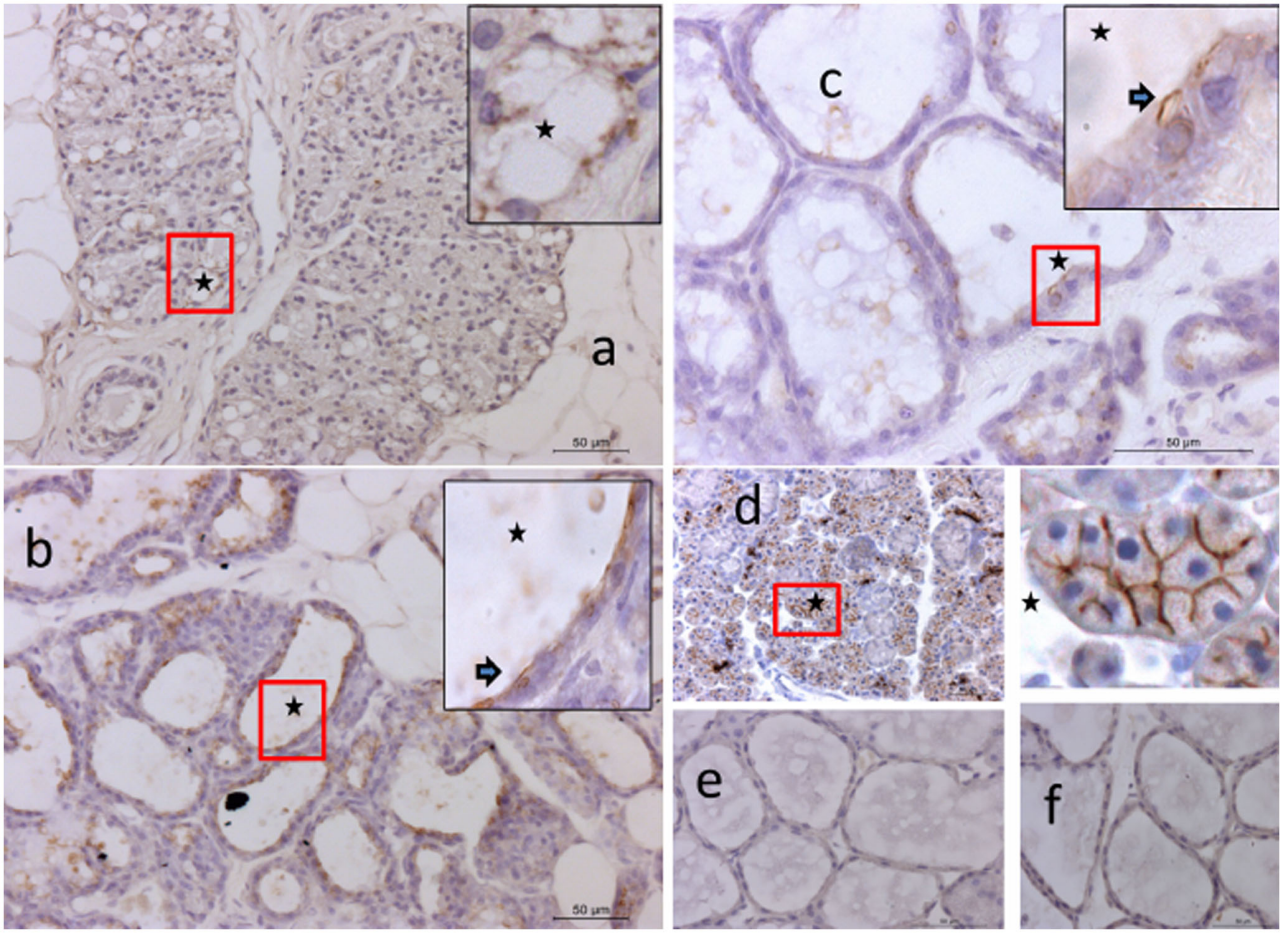
Immunostaining of rat mammary gland for AQP5 in three groups. Panel a) immuno staining of developing alveoli, pregnant group (20X). Panel b) immuno staining Early lactation group (20X) and staining towards (inset 40X indicated by the arrow), apical membrane. Panel c) Peak lactation group (20X), immune staining towards apical membrane (higher magnification 40X indicated by the arrow). Panel d) Positive control in submandibular gland of rat, staining at mucus acini (both in low magnification 20X and 40X). Panel e) Negative control during early lactation (20X). Panel f) Isotype control, IgG during early lactation (20X).

Specific staining of all three AQP was detected at all stages. AQP1 was visualized during pregnancy in the expanding alveoli (Figure 3a), during early lactation both at the secretory epithelium and blood vessels (Figure 3b) and during peak lactation primarily associated with blood vessels (Figure 3c). AQP3 was detected during pregnancy but appeared less intense than AQP1 (Fig. 4a). During lactation, AQP3 was clearly associated with alveoli, and especially the basolateral aspect of secretory cells (Fig. 4b). Often, the localization was specific to the lateral membrane (Fig. 4c). AQP5 staining intensity was lower than for the other AQP, but was clearly associated with groups of developing and secreting alveoli (Fig. 5). During lactation, AQP5 was mainly detected at the apical membrane of the alveoli (Fig. 5b, 5c). AQP5 staining was more prominent during early lactation than at peak lactation.

## Discussion

Our results provide the first evidence of developmental regulation of AQP expression in the mammary gland, and reveal a different pattern for AQP1 and AQP3 (up-regulated during lactation) compared to AQP5 (down-regulated). Tissue localization also differed. Secretory cells mainly expressed AQP3 basolaterally and AQP5 apically, whilst AQP1 was associated with alveoli and also blood vessels.

In the sense of expression being primarily present only in tissues that have a major role in transporting or retaining water, developmental regulation of aquaporin expression is common [5]. However, in the majority of cases this requirement and hence regulation is more or less constant across the lifetime of the animal, thus developmental regulation does not occur in a chronological sense. There are exceptions. In the reproductive tract, AQP expression varies during the course of the oestrous cycle, at least in rats [17] and horses [18]. Salivary glands secrete rapidly and intermittently in response to muscarinic or adrenergic agonists, but the expression of salivary AQP does not exhibit developmental regulation. Rather, there is evidence that trafficking of AQP5 between intracellular vesicles and apical membranes is responsible for the intermittent flux (reviewed by Delporte and Steinfeld, 2006 [19]). A similar situation exists in kidney collecting ducts, where vasopressin stimulates translocation of AQP2 to the apical membrane [20]. The sudden requirement of the mammary gland to increase water flux and then maintain a high and rather constant flux for an extended period is rather unique. Our data support a potential role for AQP5 inasmuch as apical localization was observed, but from a temporal point of view the decreased expression of AQP5 between pregnancy and lactation appears to argue against a major role. Previous analyses of mammary tissue have failed to detect AQP5 expression in secretory cells and Matsuzaki *et al* [12] have suggested that the constant nature of milk secretion removes any requirement for bulk flow, such that AQP expression is not required for secretion of milk. Given the very large volumes of milk that are secreted, it seems unlikely that there is no involvement of water channels. It may be that the AQP responsible for this flux is still to be identified. On the other hand, we did observe AQP5 localized to the apical membrane, and there may be another explanation for the paradoxical developmental expression pattern. AQPs are extremely stable proteins, hence, it is entirely possible that the higher AQP5 mRNA expression during pregnancy represents the time at which the protein is synthesized, concomitant with secretory cell proliferation, and it then acquires and subsequently retains functionality after parturition. From a regulatory point of view, there is evidence that the mammogenic steroids oestrogen and progesterone stimulate AQP5 expression in uterine epithelial cells of ovariectomized rats [21], hence the synthesis of mammary AQP5 may be under the control of oestrogen and progesterone during pregnancy. During lactation, prolactin is essential for the maintenance of secretory activity in many species, including rats. Interestingly, in the lacrimal gland the translocation of AQP5 to the apical membrane during tear secretion involves binding to the prolactin-inducible protein (PIP) [22]. The specific role of prolactin in the mammary exocytosis process remains elusive, although a role of SNARE (Soluble N-ethylmaleimide-Sensitive Factor Attachment Protein Receptor) proteins has been suggested [23]. Although primarily associated with metastatic mammary cells, PIP is also found in milk [24] and is sometimes used as a marker of apocrine secretion. Hence, prolactin may regulate water flux into milk through up-regulating PIP and hence translocation of AQP5. It is important to remember that this translocation may not be direct to the apical membrane. Water first enters the Golgi vesicle, and it will be important in future studies to identify whether AQP5 (or other AQP) are present on the Golgi (the immunohistochemistry procedures used here would not have detected AQP on intracellular membranes). In addition to variation in expression and translocation, it has also been proposed that water flux may be regulated by gating of AQPs (reviewed by Sachdeva and Singh, 2014 [25]). Structurally, AQP5 can be shown to possess the necessary characteristics for computer-simulated gating [26]. The whole area of AQP gating remains somewhat controversial, and since gating of AQP5 has not been shown to occur *in* *vivo* it will not be discussed further here.

In agreement with previous observations in mammary tissue, we observed AQP1 mainly localized to the capillaries. However, the most abundant AQP expression and largest increase that we observed during lactation was associated with APQ3, which localized to the basolateral (ie uptake) membrane of secretory cells. This also agrees with previous observations in mouse and bovine [12], [13]. Western blotting confirmed significant presence of AQP3 throughout lactation. Lactating mammary tissue synthesizes and secretes large amounts of triglyceride [27], and as a consequence has a significant requirement for glycerol. Several AQP (the aquaglyceroporins) including AQP3 selectively transport glycerol in addition to water. In adipose tissue and liver the predominant glycerol transporters are AQP7 and AQP9, respectively [28]. There is limited evidence of expression of mRNA for both AQP7 and AQP9 in lactating rat mammary tissue [12] but no attempt has been made to detect the proteins themselves. AQP7 appears to be regulated by insulin in a negative fashion, implying that is more involved with efflux of glycerol from hepatocytes during lipolysis (eg in starvation) than with influx during adipogenesis, and in this context the mammary gland (which is insulin insensitive) would be unlikely to have a requirement for AQP7. AQP3, on the other hand, does have a potential role in mammary glycerol uptake and this could explain the high amount of this protein located at the basolateral membrane of the secretory cells. We observed preferential localisation of AQP3 to the lateral membrane of some secretory cells. The reason for this is unclear, but could be related to localized differences in extracellular fluid substrate concentration. The contraction of myoepithelial cells and hence secretory alveoli during milk ejection will induce considerable movement of extracellular fluid in the vicinity of the alveoli, but the consequences are unknown.

The importance of mammary AQP expression for physiological as well as pathological mammary function has recently been reviewed [29]. This includes discussion of the possible role of AQP in “diluting” milk during storage within the duct system. It is important to recognize that the accepted mechanism for regulating the composition of the aqueous phase of milk is based on the principle that milk, at the point of secretion by the epithelial cell, has an osmolarity that is virtually identical to plasma [3]. Thus, although the duct system can be shown to be permeable to water (and expresses AQPs), there will be little if any net flux of water into milk after it is secreted. Although ours is the first demonstration of developmental regulation of AQP expression in mammalian lactation, a recent publication has documented developmentally regulated expression of AQP during the reproductive cycle of the Tsetse fly [30]. This viviparous insect produces a milk-like secretion from an accessory uterine milk-gland to nourish the single larva during its intrauterine development. Milk-gland AQP expression increases during this “lactation” phase, and knock-down of expression results in milk of increased osmolarity, indicative of disturbed water flux [30].

In conclusion, we have confirmed the presence in rat mammary tissue of AQP1, 3 and 5, and have demonstrated developmental regulation of all three. A role for AQP5 in regulated water secretion into milk and for AQP3 in uptake of glycerol can be proposed.
